# Effects of activated vitamin D, alfacalcidol, and low-intensity aerobic exercise on osteopenia and muscle atrophy in type 2 diabetes mellitus model rats

**DOI:** 10.1371/journal.pone.0204857

**Published:** 2018-10-17

**Authors:** Manabu Akagawa, Naohisa Miyakoshi, Yuji Kasukawa, Yuichi Ono, Yusuke Yuasa, Itsuki Nagahata, Chiaki Sato, Hiroyuki Tsuchie, Hiroyuki Nagasawa, Michio Hongo, Yoichi Shimada

**Affiliations:** Department of Orthopedic Surgery, Akita University Graduate School of Medicine, Akita City, Akita, Japan; Max Delbruck Centrum fur Molekulare Medizin Berlin Buch, GERMANY

## Abstract

Diabetes mellitus causes secondary osteoporosis and muscle atrophy. The ability of alfacalcidol (ALF) and exercise (Exe) to inhibit osteoporosis and muscle atrophy in type 2 diabetes mellitus (T2DM) model rats was examined. Twenty-week-old Otsuka Long-Evans Tokushima Fatty rats were randomized to ALF (orally 0.1 μg/kg/day), Exe (treadmill exercise at 10 m/min, 60 min/day, 5 days/week), Comb (ALF and Exe), and Cont (T2DM control treated with vehicle and no exercise) groups (n = 8–10 per group). Sedentary Long-Evans Tokushima Otsuka rats were used as a non-hyperphagic control. After treatment for 2 or 6 weeks, blood glucose (BG) levels, cross-sectional area (CSA) of tibialis anterior muscle fibers, femoral bone mineral density (BMD), and relative quantities of muscle anabolic markers (*Pax7*, *MyoD*, and *myogenin*) and catabolic markers (*Atrogin-1*, *MuRF1*, and *REDD1*) of the soleus muscle assessed by real-time polymerase chain reaction assays were measured. Exe and Comb treatments for 6 weeks decreased BG levels compared with those of the Cont group. ALF, Exe, and Comb treatments for 2 and 6 weeks recovered the CSA compared with that of the Cont group. ALF and Comb treatments for 6 weeks increased femoral BMDs compared with those of the Cont group. After 2 weeks of treatment, Comb treatment increased *MyoD* expression and decreased *MuRF1* expression. ALF or Exe monotherapy significantly decreased *Atrogin-1* or *MuRF1* expression after 2 weeks of treatment, respectively. After 6 weeks of treatment, ALF and Comb treatments decreased *Atrogin-1* and *REDD1*. These results demonstrate that a combination of ALF and Exe improved CSA from the early phase of treatment by stimulating skeletal muscle differentiation and suppressing muscle catabolic genes. Improvements in BG, BMD, and CSA were observed as long-term effects of the combination therapy. Continued suppression of muscle catabolic genes was observed as a background to these effects.

## Introduction

In recent years, population aging has become a global public health issue. Aging societies face a number of issues, such as an increase in aging-associated diseases, multiple comorbidities, and decrease in activities of daily living (ADLs) of elderly persons, who eventually become bedridden. In addition, the prevalence of age-related musculoskeletal disorders, especially osteoporosis and sarcopenia, is also increasing. Several recent reports demonstrated that osteoporotic fractures directly decrease ADLs of elderly persons, as well as increase their mortality risk [1–3]. Sarcopenia also decreases ADLs of elderly persons [4] and is strongly correlated with osteoporosis [5–8]. Therefore, in aging societies, management of aging-associated diseases, osteoporosis and sarcopenia, is imperative to maintain ADLs and improve the life expectancy of elderly persons.

Diabetes mellitus (DM) is one of the most common aging-associated diseases. A recent report showed that 8.4% of global all-cause mortality could be attributed to DM [9]. DM is associated with not only mortality, but also a decrease in activity of patients. Recent meta-analyses showed that patients with type 2 DM (T2DM), which accounts for most cases of DM, have an increased risk of fracture, even though they have normal or high bone mineral density (BMD) [10, 11]. T2DM is associated with decreased balance and increased risk of falls [12, 13]. Furthermore, T2DM patients have an increased risk of muscle atrophy, especially elderly patients [14, 15]. Thus, elderly T2DM patients have a very high risk of fracture, which is directly associated with a reduction in ADLs.

Vitamin D is a fundamental drug for the treatment of osteoporosis. A meta-analysis showed that both native and activated vitamin D prevent osteoporosis and reduce the risk of falls [16]. Alfacalcidol, activated vitamin D, also increased muscle strength in ovariectomized and glucocorticoid-treated rats [17, 18]. An in vivo study showed that 1,25-dihydroxyvitamin D3 stimulated the expression of muscle anabolic markers as a mechanism of the beneficial effects on muscle [19]. Vitamin D is essential for calcium and phosphorus homeostasis, and it also has an important role in controlling blood glucose (BG) levels. Several papers have reported the interaction between vitamin D and DM. Vitamin D deficiency inhibited insulin secretion by 48% [20], and there was an inverse association between serum 25-hydroxy vitamin D [25(OH)D] and the onset of T2DM [21]. Thus, vitamin D has the potential to reduce the risk of fracture and to improve muscle atrophy and BG levels in T2DM patients.

On the other hand, exercise has been used as a basic treatment to control BG levels and prevent the complications of T2DM [22–24]. Furthermore, exercise is also important in the management of osteoporosis. Recent studies have shown the benefits of exercise, for example, increased BMD [25–27], improved balance [28–30], and fewer falls [31]. Exercise-induced mediators, such as peroxisome proliferator-activated receptor-γ coactivator 1α [32–34], Akt [35], and insulin-like growth factor-1[36], are associated with these effects. Exercise is also an important factor in preventing muscle atrophy in T2DM patients. Specific training was shown to be effective in improving gait speed, balance, muscle strength, and joint mobility in DM patients [37, 38].

Based on these findings, we hypothesized that the combination therapy of vitamin D and exercise improves BMD and muscle weakness in T2DM. Although a meta-analysis investigated the effects of vitamin D on the neuromuscular remodeling following exercise in non-diabetic controls, the effect is still inconclusive, and there has been no investigation of bone properties [39]. Furthermore, no studies have investigated the effects of the combination therapy in T2DM. Therefore, the purpose of this study was to elucidate the effects of combination therapy of activated vitamin D and exercise on bone and skeletal muscle in T2DM model rats.

## Materials and methods

### Animals and experimental protocol

**Fig 1:**
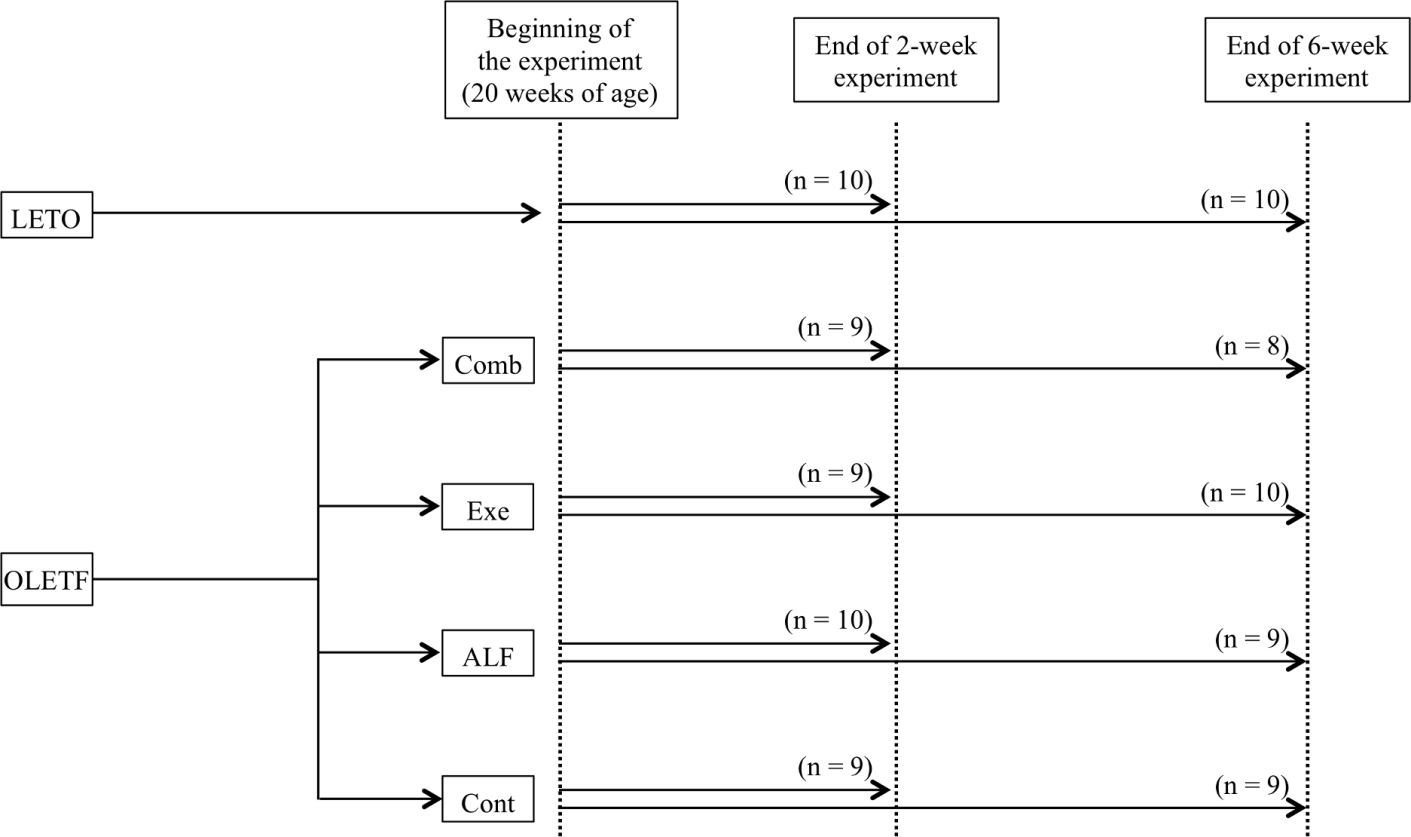
Diagram of the experimental protocol. OLETF rats were assigned to each treatment group. Each treatment began at the age of 20 weeks. OLETF = Otsuka Long-Evans Tokushima Fatty; LETO = Long-Evans Tokushima Otsuka (non-diabetic control); Cont = control (diabetic control); ALF = alfacalcidol; Exe = exercise; Comb = combination of alfacalcidol and exercise.

Six-week-old, male Otsuka Long-Evans Tokushima Fatty (OLETF) rats and Long-Evans Tokushima Otsuka (LETO) rats (Hoshino Laboratory Animals, Ibaraki, Japan) were housed in a controlled environment (temperature 23 ± 2°C, humidity 40% ± 20%) with a 12-h light-dark cycle. The details were described in the previous study [40]. OLETF rats are a model of T2DM, while LETO rats are a control model of T2DM (non-T2DM).[41] Rats were allowed *ad libitum* access to standard rodent chow (CE-7; Clea Japan, Tokyo, Japan) and remained sedentary until 20 weeks of age. OLETF rats have been reported to show increased levels of blood glucose from 20 weeks of age [41]. OLETF rats were randomly assigned to one of the following groups at the age of 20 weeks (n = 8-10/group): (1) ALF group, administered alfacalcidol; (2) Exe group, low-intensity aerobic exercise training; (3) Comb group, administered alfacalcidol and low-intensity aerobic exercise training; and (4) T2DM control group (Cont group), administered vehicle. LETO rats were kept in a sedentary cage condition as non-hyperphagic controls. Each treatment began at 20 weeks of age and continued for 2 or 6 weeks (Fig 1). The following parameters were analyzed in LETO rats to check the changes of these parameters with T2DM and the treatment effects on these parameters to determine whether they recovered to the level of non-T2DM control (LETO) rats. The protocols for all animal experiments were approved in advance by the Animal Experimentation Committee at Akita University (permit No. a-1-2729), and all subsequent animal experiments adhered to the “Guidelines for Animal Experimentation” of Akita University.

### Body weight measurement

Body weight (BW) was measured at the beginning (20 weeks of age) and end of the experiment. Changes in BW were compared among and within the groups at 2 or 6 weeks.

### ALF administration and treadmill exercise

Alfacalcidol (Chugai Pharmaceutical, Tokyo, Japan) at a dose of 0.1 μg/kg/day or vehicle (medium-chain triglyceride) was administered orally every day for 2 or 6 weeks. This dose was selected based on previous studies demonstrating that it did not increase serum calcium levels [17, 18].

Low-intensity treadmill exercise was performed at a speed of 10 m/min, 5% incline, 60 min/day for 2 or 6 weeks (MK-680; Muromachi Kikai, Tokyo, Japan).

### Blood glucose measurement

Blood samples were taken from the tail vein for blood glucose measurement. BG (mg/dl) was measured using an automatic analyzer (Antsense III; Horiba, Kyoto, Japan) at the beginning (20 weeks of age) and end of the experiment. Changes in BG levels were compared among and within the groups at 2 or 6 weeks.

### Tissue preparation

Rats were euthanized by an injection of sodium pentobarbital (150 mg/kg body weight) (Dainippon Sumitomo Pharma Co. Ltd., Osaka, Japan), and the right femur, left tibialis anterior muscle, and soleus muscle were harvested for evaluations after 2 or 6 weeks of treatment.

The right femur was dipped in 10% formalin neutral buffer (Wako Pure Chemical Industries, Osaka, Japan) for BMD measurement. The left tibialis anterior muscle was immediately frozen in liquid nitrogen and stored at -80°C for histological analyses. The soleus muscle was stored in RNAlater solution (Qiagen, Hilden, Germany) at -80°C for gene expression analysis.

### BMD measurement

BMD of the femur was measured using dual-energy X-ray absorptiometry (QDR-4500 Delphi; Hologic, Bedford, MA, USA). The total length of the femur was trisected into proximal, middle, and distal regions. Each region was scanned in the “small animal” mode, with the “regional high-resolution” scan option.

### Histological analysis of muscle

The left tibialis anterior muscle was analyzed histologically. Samples were cut into 10-μm-thick transverse serial sections at the thickest part of the muscle belly using a cryostat maintained at -18°C. Sections were stained histochemically (hematoxylin and eosin, H&E).

To measure the cross-sectional area (CSA) of muscle fibers, microscopic images at a magnification of 200× were captured digitally (BX-50; Olympus, Tokyo, Japan), and individual muscle fibers were traced on-screen using ImageJ (National Institutes of Health, Bethesda, MD, USA). Areas were calculated using the ImageJ software based on a calibrated pixel-to-actual size (micrometer) ratio. Fifty fibers per muscle were randomly chosen, and the mean CSA for one muscle fiber was calculated.

Intra-observer variation, as assessed by the coefficient of variation for three corresponding measurements in 50 randomly selected fibers, ranged from 0.8% to 2.6%. Inter-observer variation among the three investigators, as assessed by the coefficient of variation of measurements in 50 randomly selected images, ranged from 2.8% to 6.3%.

### Gene expression analysis of skeletal muscle

The soleus muscle was prepared using a homogenizer (MS-100R; Tomy, Tokyo, Japan). Total RNA was extracted and reverse transcribed using an Omniscript Reverse Transcription kit (Qiagen), according to the manufacturer’s instructions. The gene expressions of the following were examined: paired box protein-7 (*Pax7*), *MyoD*, and *myogenin* as muscle anabolic markers; *Atrogin-1* and muscle ring finger 1 (*MuRF1*) as muscle catabolic markers; and regulated in development and DNA damage responses 1 (*REDD1*). The real-time polymerase chain reaction (PCR) was carried out with TaqMan probes specific for rat *Pax7* (Taqman probe ID: Rn00834076_m1), *MyoD* (Taqman probe ID: Rn01457527_g1), *myogenin* (Taqman probe ID: Rn01490689_g1), *Atrogin-1* (Taqman probe ID: Rn00591730_m1), *MuRF1* (Taqman probe ID: Rn00590197_m1), and *REDD1* (Taqman probe ID: Rn01433735_g1). Glyceraldehyde-3-phosphate dehydrogenase (GAPDH) was used as an internal control for sample normalization (Taqman probe ID: Rn01775763_g1).

### Statistical analyses

All data are expressed as means ± standard deviation (SD). The results of gene expression did not show a normal distribution; therefore, the nonparametric gene expression data were analyzed using the Kruskal-Wallis test and Dunn’s method as a post hoc test. All other data were parametric and analyzed using one-way analysis of variance (ANOVA) and Scheffé’s post hoc test. The paired *t*-test was used for the analyses of body weight and BG changes within the groups. All statistical analyses were performed using Statistical Package for the Biosciences Software (SPBS v 9.6) [42]. Values of *p* < 0.05 were considered significant.

## Results

### Combined treatment with ALF and exercise prevented the increase of body weight

The BW of LETO rats was significantly lower than of the OLETF groups at any time point (*p* < 0.0001, respectively). There were no significant differences between the OLETF groups at any time points.

Comparing BW before and after treatment within the groups, the BWs of the Cont and LETO groups after 2 weeks of treatment were significantly increased from before treatment (all *p* < 0.001). After 6 weeks of treatment, only the Comb group suppressed the increase of BW, while the other groups had significant increases of BW (all *p* < 0.01; Table 1).

**Table 1:** Body weight (g) of each experimental group.

	LETO	Cont	ALF	Exe	Comb	ANOVA
**2 weeks**						
Start	476 ± 21	608 ± 23*	600 ± 41*	595 ± 29*	604 ± 28*	*p* < 0.0001
Sacrifice	490 ± 23^†^	622 ± 26^†^*	600 ± 38*	594 ± 30*	586 ± 40*	*p* < 0.0001
**6 weeks**						
Start	488 ± 20	599 ± 32*	602 ± 20*	602 ± 28*	601 ± 17*	*p* < 0.0001
Sacrifice	518 ± 18^†^	636 ± 37^†^*	627 ± 27^†^*	624 ± 31^†^*	606 ± 21*	*p* < 0.0001

mean ± SD

n = 8–10 in each group

*: *p* < 0.01 vs. LETO group by Scheffé’s method

†: significantly increased from the start of the experiment within the group

### Exercise and combined treatment with ALF and exercise decreased blood glucose levels

Although BG levels did not differ significantly among the groups at the start of the experiment and after 2 weeks of treatment, the BG level of T2DM model rats (Cont group) was significantly higher than that of non-T2DM rats (LETO group) (*p* < 0.001) after 6 weeks. Exercise and combined treatment with exercise and ALF significantly decreased BG levels (all *p* < 0.05) compared with that of the Cont group after 6 weeks of treatment.

Comparing BG levels before and after treatment within the groups, the BG levels of the Cont group after 2 and 6 weeks of treatment were significantly increased from before treatment (*p* = 0.00245, *p* = 0.0083, respectively; Table 2). In the Exe and Comb groups, the BG level tended to decrease compared to that at the start after 6 weeks of treatment, but not significantly.

**Table 2:** Blood glucose level (mg/dL) of each experimental group.

	LETO	Cont	ALF	Exe	Comb	ANOVA
**2 weeks**						
Start	169.7 ± 18.0	181.9 ± 17.1	180.7 ± 18.4	214.2 ± 45.7	213.2 ± 45.1	
Sacrifice	157.1 ± 19.7	225.6 ± 39.6^†^	198.2 ± 23.3	220.7 ± 60.0	206.0 ± 60.2	*p* = 0.0655
**6 weeks**						
Start	172.1 ± 11.4	211.6 ± 41.9	206.3 ± 25.6	203.1 ± 33.5	215.3 ± 52.4	
Sacrifice	167.5 ± 22.1*	288.0 ± 87.2^†^	239.8 ± 39.6	191.9 ± 29.3*	204.6 ± 40.8**	*p* = 0.0001

n = 8–10 in each group

*: *p* < 0.01 vs. Cont group by Scheffé’s method

**: *p* < 0.05 vs. Cont group by Scheffé’s method

†: significantly increased from the start of the experiment within the group

### Both ALF monotherapy and combined treatment with ALF and exercise increased femoral BMD

**Fig 2:**
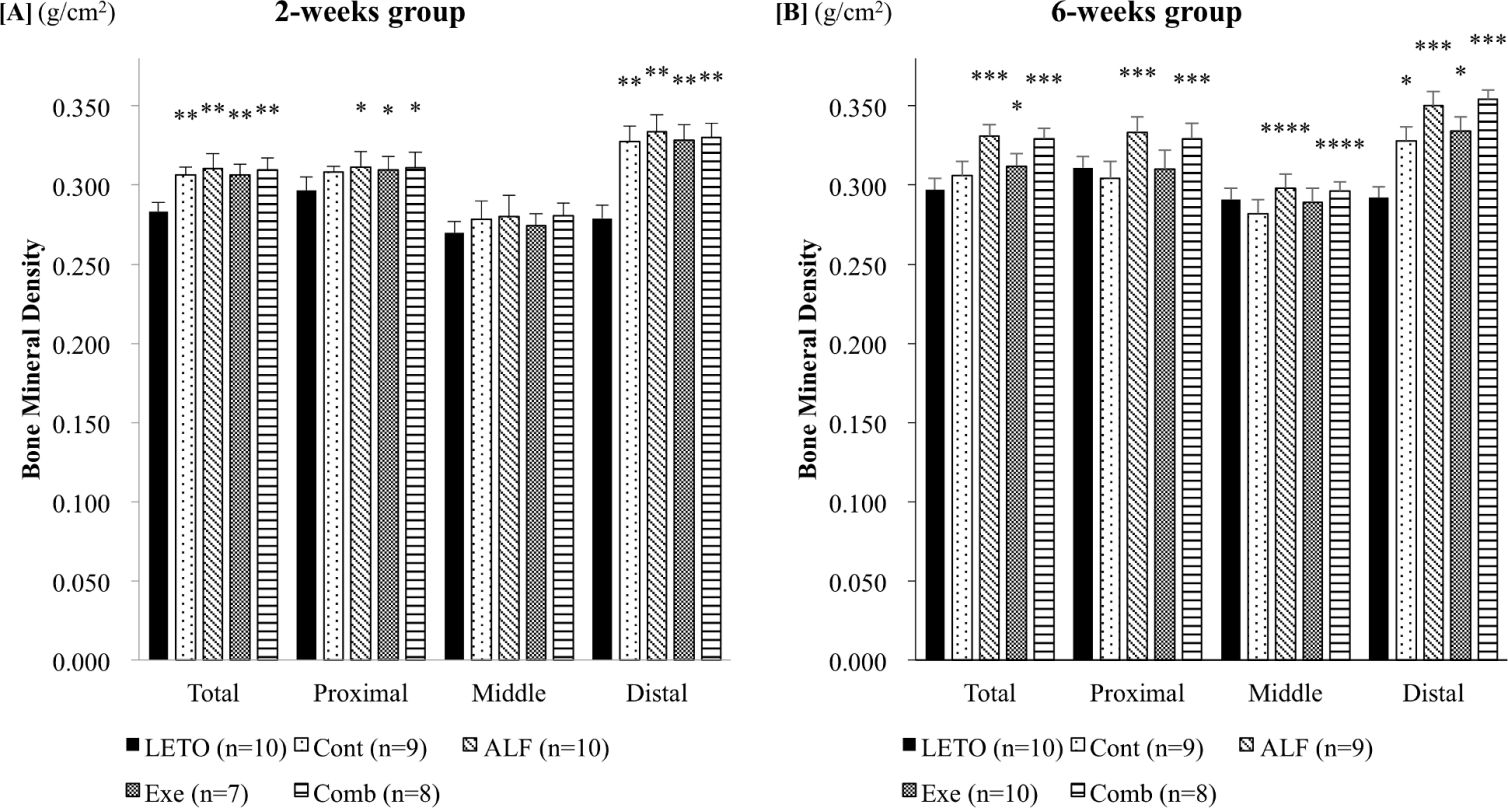
Bone mineral density of the femur. [A] 2-week group BMD values in T2DM model rats (Cont, ALF, Ex, and Comb groups) are significantly higher than those in the non-T2DM rats (LETO group). However, there is no significant difference in cortical (middle femoral) BMD among the groups. [B] 6-week group Both ALF monotherapy and combined treatment with ALF and exercise increase femoral BMD. *: *p* < 0.05 vs. LETO group by Scheffé’s method **: *p* < 0.0001 vs. LETO group by Scheffé’s method ***: *p* < 0.05 vs. LETO, Cont, and Exe groups by Scheffé’s method ****: *p* < 0.05 vs. Cont group by Scheffé’s method LETO = Long-Evans Tokushima Otsuka (non-diabetic control); Cont = control (diabetic control); ALF = alfacalcidol; Exe = exercise; Comb = combination of alfacalcidol and exercise.

Total and distal femoral BMDs of T2DM model rats (Cont groups) were significantly higher than of non-T2DM rats (LETO group, all *p* < 0.0001) after 2 weeks of treatment. Two weeks of treatment with ALF with or without exercise significantly increased the total, proximal, and distal femoral BMDs compared with those of non-T2DM rats (LETO group, all *p* < 0.05 - < 0.0001; Fig 2A), but not with T2DM control rats (Cont group). However, there was no significant difference in cortical (middle femoral) BMD among the groups. ALF and combined treatment with ALF and exercise for 6 weeks significantly increased the total, proximal, and distal femoral BMDs compared with those of T2DM control rats (Cont group), non-T2DM rats (LETO group), and rats treated with only exercise (Exe group) (all *p* < 0.05). ALF and combined treatment significantly increased cortical (middle femoral) BMD compared with that of T2DM control rats (Cont group, all *p* < 0.05). Treadmill exercise significantly increased total and distal femoral BMDs compared with those of non-T2DM rats (LETO group, all *p* < 0.05). Distal femoral BMD was significantly higher in T2DM control rats (Cont group) than in non-T2DM rats (LETO group, *p* < 0.05; Fig 2B).

### Combined treatment with ALF and exercise for 2 weeks recovered CSA to that of non-T2DM rats in the tibialis anterior muscle

**Fig 3:**
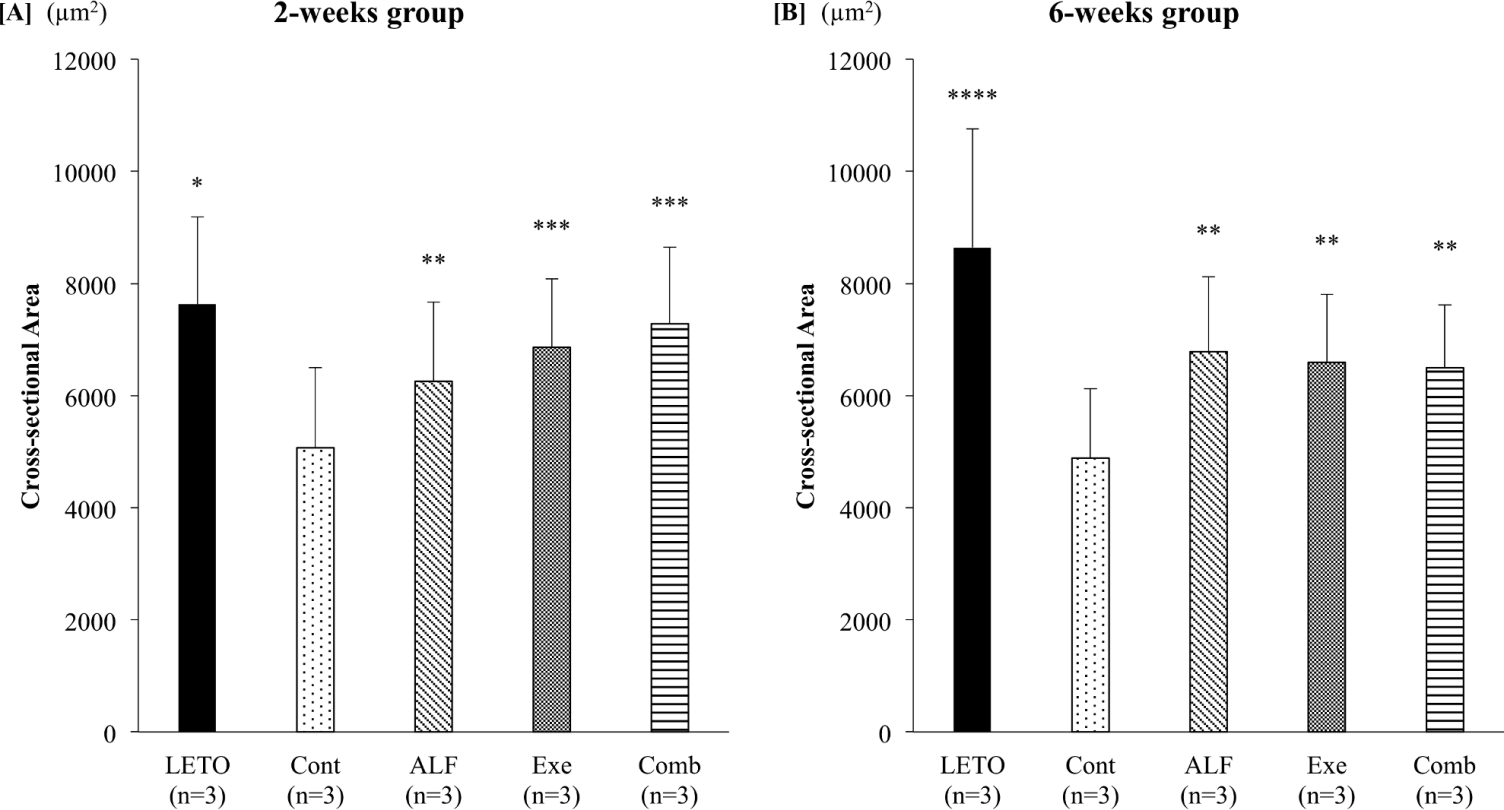
Cross-sectional area of the tibialis anterior muscle. [A] 2-week group Only combined treatment with ALF and treadmill exercise for 2 weeks recovers the CSA to that of non-T2DM rats. [B] 6-week group ALF, treadmill exercise, and combined treatment significantly increase the CSA compared with that of T2DM control rats. *: *p* < 0.01 vs. Cont, ALF, and Ex groups by Scheffé’s method **: *p* < 0.01 vs. Cont group by Scheffé’s method ***: *p* < 0.01 vs. Cont and ALF groups by Scheffé’s method ****: *p* < 0.01 vs. Cont, ALF, Exe, and Comb groups by Scheffé’s method LETO = Long-Evans Tokushima Otsuka (non-diabetic control); Cont = control (diabetic control); ALF = alfacalcidol; Exe = exercise; Comb = combination of alfacalcidol and exercise.

ALF and/or treadmill exercise for 2 weeks significantly increased the CSA compared with that of T2DM control rats (Cont group, all *p* < 0.01). Although monotherapy with ALF or treadmill exercise for 2 weeks could not recover the CSA to that of non-T2DM control rats (LETO), combined treatment with ALF and exercise significantly increased the CSA compared with that of ALF monotherapy (*p* < 0.01) and significantly recovered the CSA to that of non-T2DM control rats (LETO group). Exercise monotherapy significantly increased CSA compared with ALF monotherapy (*p* < 0.01) (Fig 3A).

After 6 weeks of treatment, ALF, treadmill exercise, and combined treatment significantly increased the CSA compared with that of T2DM control rats (Cont group, all *p* < 0.01); however, the CSAs in those groups were still significantly smaller than of non-T2DM rats (LETO group, all *p* < 0.01; Fig 3B).

### Combined treatment with ALF and exercise increased MyoD expression and decreased MuRF1 expression of the soleus muscle

**Fig 4:**
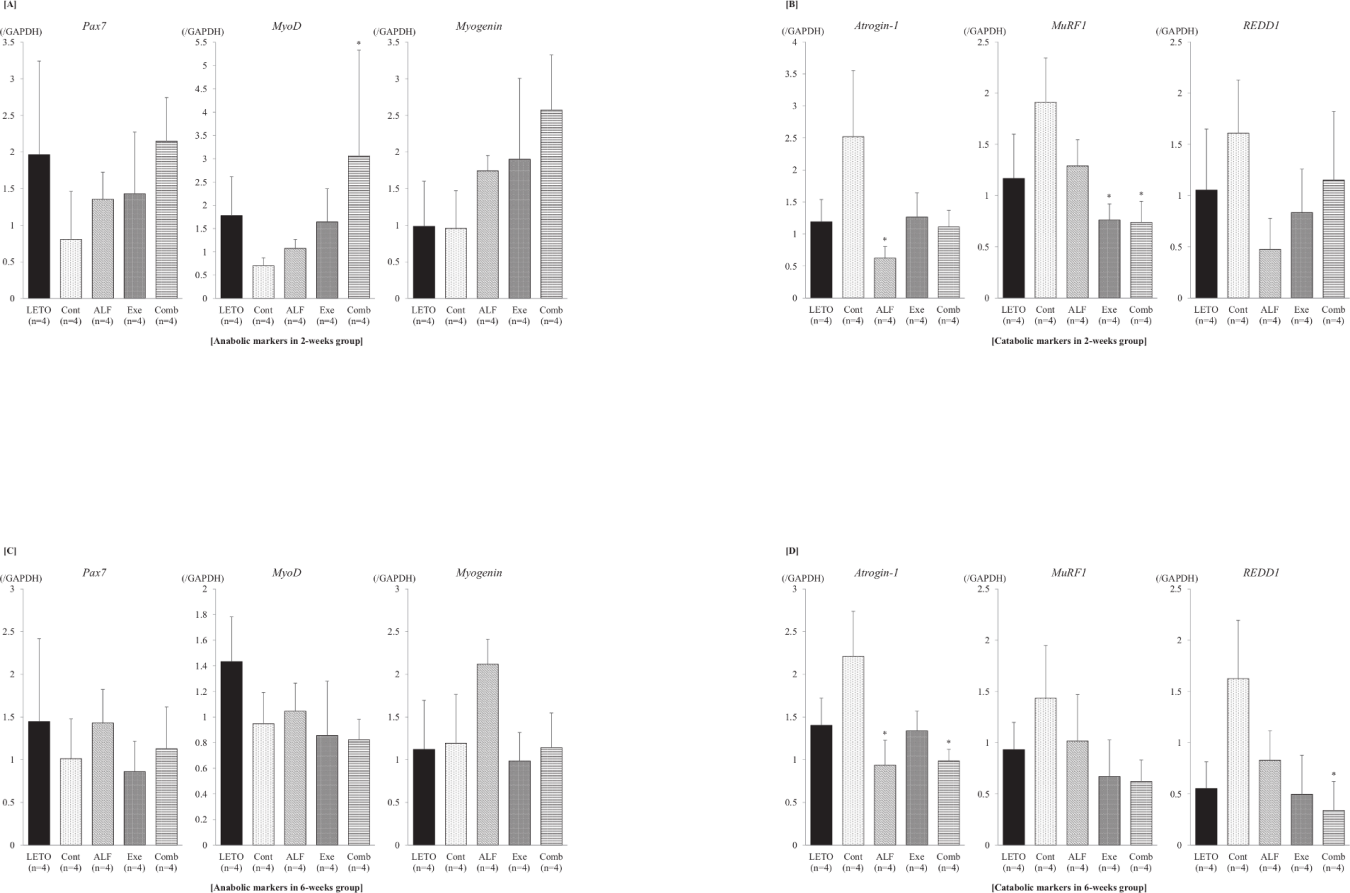
Gene expressions of muscle anabolic and catabolic markers. [A] Muscle anabolic markers in the 2-week group Combined treatment with ALF and treadmill exercise increases *MyoD* expression. [B] Muscle catabolic markers in the 2-week group Combined treatment with ALF and treadmill exercise decreases *MuRF1* expression, and ALF or treadmill exercise monotherapy for 2 weeks significantly decreases *Atrogin-1* or *MuRF1* expression compared with that of T2DM control rats. [C] Muscle anabolic markers in the 6-week group There are no significant differences among the groups. [D] Muscle catabolic markers in 6-weeks group Combined treatment with ALF and treadmill exercise significantly decreases *Atrogin-1* and *REDD1* expressions compared with that of T2DM control rats. ALF monotherapy also significantly decreases *Atrogin-1* expression. *: *p* < 0.05 vs. Cont group by Dunn’s method LETO = Long-Evans Tokushima Otsuka (non-diabetic control); Cont = control (diabetic control); ALF = alfacalcidol; Exe = exercise; Comb = combination of alfacalcidol and exercise.

Combined treatment with ALF and treadmill exercise for 2 weeks significantly increased *MyoD* expression (Fig 4A) and decreased *MuRF1* expression (Fig 4B) compared with those of T2DM control rats (Cont group, all *p* < 0.05). ALF or treadmill exercise monotherapy for 2 weeks significantly decreased the expression of *Atrogin-1* (*p* < 0.05) or *MuRF1* (*p* < 0.05) compared with that of T2DM control rats (Cont group)(Fig 4B).

After 6 weeks of treatment, there were no significant differences in muscle anabolic markers (*Pax7*, *MyoD*, and *myogenin*) among the groups (Fig 4C). On the other hand, combined treatment with ALF and treadmill exercise significantly decreased *Atrogin-1* and *REDD1* expressions compared with those of T2DM control rats (Cont group, all *p* < 0.05). ALF monotherapy also significantly decreased *Atrogin-1* expression (*p* < 0.05; Fig 4D).

## Discussion

In this study, T2DM model rats (OLEFT rats) showed increased total and distal femoral BMDs at 2 weeks and decreased CSA of the tibialis anterior muscle at 2 and 6 weeks compared with non-T2DM control rats (LETO rats). However, there were no significant changes of muscle anabolic and catabolic-related gene expressions in the soleus muscle compared with those of non-T2DM control rats (LETO rats). The effects of combination treatment with ALF and treadmill exercise on bone and skeletal muscle were examined in T2DM model rats. As a short-term effect, combination therapy enhanced muscle anabolic marker (*MyoD)* expression and recovered the CSA of the tibialis anterior muscle compared with T2DM control rats. As a long-term effect, combined treatment significantly increased femoral BMDs, decreased BG levels, and suppressed muscle catabolic marker (*REDD1*) expression.

### Effect of ALF on blood glucose levels

ALF monotherapy did not inhibit the increase in BG levels in T2DM rats in the present study. Several previous studies have documented the relationship between vitamin D and BG levels or insulin resistance in subjects with lower serum vitamin D, 25(OH)D, levels [43]. However, recent meta-analyses have shown that there is insufficient evidence of a beneficial effect of vitamin D supplementation on improving glycemia or insulin resistance in patients with diabetes, normal fasting glucose, or impaired glucose tolerance [44, 45]. Further investigation is likely needed to elucidate the effects of vitamin D on BG levels or insulin resistance, especially in patients with vitamin D deficiency and T2DM.

### BMD in T2DM

A meta-analysis has demonstrated that T2DM is a risk factor for proximal femoral fractures compared to patients without DM [46]. However, a decrease in BMD is not necessarily seen in T2DM when compared with non-DM, even though the risk of fractures is higher [11, 47]. Distal metaphyseal femoral BMD in OLETF rats as a model of T2DM was also increased compared with the control T2DM (LETO) rats in the present study. On the other hand, cortical (middle femoral) BMD in OLETF rats did not show a significant change compared with LETO rats. A previous study demonstrated that cortical BMD of the tibia, measured by peripheral quantitative computed tomography, at 20 weeks was significantly higher in OLETF rats than in LETO rats [48]. Differences in the measurement equipment used or the site selected for cortical BMD determination may have contributed to the difference in cortical BMD in T2DM OLETF rats.

### Effects of ALF and treadmill exercise on BMD in T2DM

In T2DM model rats, 6 weeks of treatment with ALF increased proximal femoral BMD. A randomized, placebo-controlled study reported that ALF increased BMD of the lumbar spine by 2.3% in osteoporotic women [49]. However, few studies have investigated the effect of ALF on BMD in T2DM.

On the other hand, treadmill exercise did not exert a significant effect on BMD in T2DM in the present study. A recent study reported that treadmill running exercise increased femoral BMD in T2DM mice [50]. Another study showed that moderate treadmill running increased femoral BMD in diabetic obese Zucker rats [51]. The duration of treadmill exercise in these studies was greater than 10 weeks. Thus, a longer duration of treadmill exercise may show significant results for BMD in T2DM model rats.

Combined treatment with ALF and treadmill exercise significantly increased the femoral BMDs, including both cancellous and cortical bone-dominant regions. This is the first study to investigate the effect of combined treatment with ALF and treadmill exercise on BMD.

### Effects of ALF and treadmill exercise on muscle

Muscle anabolic and catabolic genes were evaluated in this study. In the differentiation process of skeletal muscle, *Pax7* is essential for the activation of satellite cells [52]. *MyoD* plays a crucial role in the differentiation of satellite cells into myoblasts [53], and *myogenin* controls the differentiation of myoblasts to myotubes [54]. On the other hand, myostatin is a major autocrine inhibitor of muscle growth, and one of the important factors that promotes the expression of muscle catabolic genes such as *MuRF1* and *Atrogin-1* [55]. These two muscle-specific ubiquitin ligases are now widely used as markers of accelerated muscle atrophy [56, 57] and are related to many types of skeletal muscle atrophy. *REDD1*, which is a strong repressor of the mammalian target of rapamycin (mTOR) [58], is another important muscle catabolic gene stimulated by glucocorticoids.

In the present study, activated vitamin D and exercise monotherapy repressed *Atrogin1* and *MuRF1*, respectively. A previous report showed that activated vitamin D stimulates the myogenic differentiation process by inhibiting myostatin [59]. A recent report also showed that exercise downregulates myostatin [36]. Thus, activated vitamin D or exercise monotherapy appeared to suppress the expression of *Atrogin-1* or *MuRF1* via the downregulation of myostatin. However, each single treatment did not increase the expressions of muscle anabolic genes, and only the combination therapy increased *MyoD* expression and repressed *REDD1*.

A previous study reported that the factors associated with muscle atrophy in T2DM are increased myostatin, endogenous glucocorticoids, and insulin resistance [55].

Since activated vitamin D and exercise therapy both inhibit myostatin, as described above, it is highly likely that the combination therapy similarly suppressed myostatin. Additionally, although the mechanisms are unclear, suppression of *REDD1* means that the combined treatment decreased the endogenous glucocorticoid level. While the mechanisms of these results require further clarification, combined treatment may suppress the muscle catabolic pathway multidirectionally, which finally enables the increase of *MyoD*.

As a limitation, treadmill exercise was performed at a speed of 10 m/min. This setting is relatively low compared to that of a previous report [60]. However, the appropriate exercise setting differs depending on the type of animal model and the age of the animal. Alterations in the speed or duration of treadmill exercise may demonstrate other effects on BMD or muscle properties and related gene expressions.

In conclusion, the combination of ALF and exercise improved the CSA of the tibialis anterior muscle from the early phase (2 weeks) of treatment by stimulating *MyoD* expression and suppressing atrogenes. Improvements in BG levels, BMD, and muscle volume were observed as long-term effects of the combination therapy. Continued suppression of atrogenes and *REDD1* was observed as a background to these long-term effects. BMD and muscle-related results of this study indicate that combined treatment is recommended for the treatment of osteoporosis and muscle atrophy caused by T2DM.

The results of this study are expected to contribute to the treatment of muscle wasting and osteoporosis, which will lead to a reduction of the fracture risk and an improvement of ADLs in patients with T2DM. Since no previous reports investigated the effects of combination therapy with activated vitamin D and exercise on blood glucose levels, bone, and skeletal muscle in patients with T2DM, we would like to apply the results of the present study to clinical research and establish clinical evidence in the future study.

## Supporting information

S1 File Body weight. (XLSX)

S2 File Blood glucose level. (XLSX)

S3 File Bone mineral density. (XLSX)

S4 File Cross sectional area of the tibialis anterior muscle. (XLSX)

S5 File Gene expression of muscle anabolic and catabolic markers. (XLSX)
